# Enhancing wheat β-glucan content through precision crossbreeding: development and evaluation of biofortified lines with improved nutritional and agronomic traits

**DOI:** 10.3389/fgene.2025.1532956

**Published:** 2025-03-19

**Authors:** Upendra Kumar, Sourav Panigrahi, Rita Goswami, Yogita Singh, Priyanka Balyan, Prexha Kapoor, Sundip Kumar, Krishna Pal Singh, Farkhandah Jan, Reyazul Rouf Mir

**Affiliations:** ^1^ Department of Molecular Biology and Biotechnology, College of Biotechnology, CCS Haryana Agricultural University, Hisar, India; ^2^ Department of Plant Science, Mahatma Jyotiba Phule Rohilkhand University, Bareilly, India; ^3^ Department of Botany, Deva Nagri P.G. College, CCS University Meerut, Meerut, India; ^4^ Molecular Cytogenetics Laboratory, Department of Molecular Biology and Genetic Engineering, College of Basic Sciences and Humanities, GB Pant University of Agriculture and Technology, Pantnagar, India; ^5^ Biophysics Unit, College of Basic Sciences and Humanities, GB Pant University of Agriculture and Technology, Pantnagar, India; ^6^ Vice-Chancellor’s Secretariat, Mahatma Jyotiba Phule Rohilkhand University, Bareilly, India; ^7^ Division of Genetics and Plant Breeding, Sher-e-Kashmir University of Agricultural Sciences and Technology of Kashmir (SKUAST-Kashmir), Srinagar, India

**Keywords:** β-glucan, introgression, Aegilops kotschyi, *in situ* hybridization, SSR, backcross

## Abstract

**Introduction:** To address the urgent demand for biofortified wheat enriched with health-beneficial dietary fibres such as β-glucan, this study employed meticulous crossbreeding between established wheat cultivars and the β-glucan-rich wild relative *Aegilops kotschyi* accession “AK-3790”.

**Methods: **Within this context, a derivative line encompassing a pair of 7U chromosomes from *Ae. Kotschyi*, denoted as 63-2-13, was identified. The presence of the 7U chromosome in this line was confirmed through comprehensive molecular marker and genomic *in situ* hybridization (GISH) analyses. With the aim of increasing the β-glucan content in hexaploid wheat, two distinct backcross populations were developed utilizing the 63-2-13 line as the donor parent and two separate recurrent parents (WH1105 and HD3086). These populations underwent an exact selection regimen, encompassing parent-like phenotypes, heightened yield, and robust resistance to yellow rust, meticulously tracked across successive generations until the BC_2_F_2:3_ stage.

**Results and Discussion: **Notably, among the outcomes, selected BC_2_F_2:3_ progenies presented remarkable increases in β-glucan levels, with a notable increase (BC_2_F_2:3_ 23-5) resulting in an impressive increase in the 1.76% grain β-glucan content. Despite a discernible reduction in yield compared with their high-yielding counterparts, BC_2_F_2:3_ 23-5 demonstrated a harmonious trait profile, encompassing heightened β-glucan content and moderate yellow rust resistance, thus positioning it as a compelling candidate for subsequent refinement endeavors. This research notably underscores the substantial potential of precise introgression strategies for increasing the β-glucan content in wheat, thereby underscoring the imperative of adept trait optimization to ensure both yield stability and nutritional enhancement.

## 1 Introduction

The contemporary emphasis on fostering healthier dietary patterns has underscored the imperative of devising nutritionally fortified food products. Among the pivotal constituents of a health-promoting diet, dietary fibre, a fundamental component abundant in plant cell walls, has garnered particular attention. As dietary fibre consumption increases, evidence has emerged linking it to a reduced susceptibility to chronic maladies, including heart disease, type 2 diabetes, breast cancer, and colon cancer (Reynolds et al., 2019). Notably, cereal-derived fibres, constituting a significant share of dietary fibre intake, have gained prominence. Within this dietary fibre landscape, 1–3,1–4 mixed-linkage β-D-glucan, a prominent soluble fibre predominantly found in cereal grains such as barley and oats, has garnered special interest. This fiber exerts a multifaceted influence by mitigating risk factors associated with type 2 diabetes and cardiovascular diseases, primarily by modulating glycemic responses, reducing cholesterol levels, and attenuating insulin-related effects (Wood, 2007; Sibakov et al., 2013; Cui and Liu, 2013).

Among the diverse array of cereal crops, wheat (*Triticum aestivum* L.) plays a pivotal role as a global staple, substantially contributing to daily caloric and protein intake (FAO, 2020). However, the content of soluble β-glucans in wheat grains is notably modest, particularly in comparison to the more abundant concentrations present in oat and barley grains (Beresford and Stone, 1983; Havrlentová and Kraic, 2006; Collins et al., 2010). Despite dedicated endeavors, the primary and secondary gene pools of bread wheat have exhibited constrained genetic diversity conducive to the expression of high β-glucan contents (Pritchard et al., 2011; Marcotuli et al., 2017).

Moreover, neither bread wheat (*T. aestivum* L. 2n = 6x = 42) nor its closest relatives exhibit genetic diversity for high grain β-glucan levels (Friebe et al., 1996). This scarcity of genetic diversity extends to both the primary and secondary gene pools, limiting the feasibility of establishing a breeding program aimed at increasing the β-glucan content in cultivated wheat varieties. This conclusion stems from comprehensive studies involving over 500 wheat germplasms of diverse origins, including primitive, elite, and synthetic lines of hexaploid, tetraploid, and diploid wheat, as well as related species (Pritchard et al., 2011; Marcotuli et al., 2019).

To alter the quality of wheat grains, a strategic avenue involves incorporating genes from less closely related relatives, which may belong to the tertiary wheat gene pool and encompass both wild and cultivated species. This is achieved through chromosome engineering and interspecific hybridization (Cseh et al., 2011; 2013; Rakszegi et al., 2017; Danilova et al., 2018; Türkösi et al., 2018). Among these tertiary gene pool members, barley has the highest β-glucan concentration (up to 11%), making it an effective candidate for interspecific hybridization. Notably, certain species of Aegilops, which are also part of the wheat tertiary gene pool, present significant β-glucan contents (up to 7.1%) in their grains. These species include diploid types such as *Ae. Umbellulata* (Zhuk.), as well as various tetraploid species containing U and C genomes (Rakszegi et al., 2017; Marcotuli et al., 2019). In contrast, most bread wheat (*T. aestivum* L.) cultivars and durum wheat (*Triticum turgidum* L. ssp. durum) exhibit lower levels of β-glucan in their grains (Marcotuli et al., 2016; Mohebbi et al., 2018).

The practice of harnessing genetic traits from distantly related species to augment wheat’s genetic robustness and resilience has been previously demonstrated, notably in the context of conferring resistance against various biotic stresses (Friebe et al., 1996; 2000; Marais et al., 2005; Qi et al., 2007; Kuraparthy et al., 2007a; b; Schneider et al., 2008). In this study, we present a comprehensive exploration of the process and molecular insights underlying the successful introgression of *Ae. Kotschyi* chromosomes, thereby paving the way for an enriched β-glucan content in wheat grains.

## 2 Materials and methods

### 2.1 Plant materials

The *Ae. Kotschyi* accession AK-3790 was chosen for our breeding program because of its known high grain β-glucan concentration (up to 2.4%) (Marcotuli et al., 2019). The seeds of a wheat-*Ae. kotschyi* derivative line (63-2–13) (Tiwari et al. 2010) were sourced from the Wheat Germplasm Collection at Eternal University, Baru Sahib, Himachal Pradesh, India. This derivative line resulted from a cross between *Ae. Kotschyi* (accession AK-3790) as the male ‘donor’ and the recipient Chinese spring *PhI*. To develop two backcross populations, the derivative line 63-2-13 was employed as the donor parent in a backcrossing program, utilizing the wheat cultivars WH1105 and HD3086 as recurrent parents to generate BC_2_F_1_ plants during the winter of 2020–21. BC_2_F_2_ plants were subsequently generated, followed by self-pollination and the production of BC_2_F_2:3_ lines via the single-seed descent method in 2022–23. These lines were grown via an augmented design alongside three reference checks: Chinese spring (CS), WH1105, and HD3086, following standard wheat agronomic conditions. The breeding program was carried out at the farm of the Department of Molecular Biology and Biotechnology, while subsequent generation advancement was performed in the field of Wheat and Barley Section, Chaudhary Charan Singh Haryana Agricultural University, Hisar. On the basis of the performance of the genotypes in both generations, the BC_2_F_2:3_ population underwent screening for traits such as yield, yellow rust resistance, and parental backgrounds. The seeds of BC_2_F_2:3_ lines of selected agronomical superior plants were screened for grain β-glucan content, aiming to identify progeny lines with significantly elevated β-glucan levels comparable to those of checks WH1105 and HD3086.

### 2.2 Trait phenotyping

At maturity, a range of morphological and phenotypic traits were observed and recorded in the field. These traits included plant height, the number of productive tillers, thousand-grain weight, and grain yield per plant. Additionally, the yellow rust score was documented during the anthesis stage according to the modified Cobb scale of 1-5 provided by Peterson et al. (1948), along with the severity of infection as a percentage.

### 2.3 Data analysis

Since the BC_2_F_2:3_ population is representative of the BC_2_F_2_ population, the mean data obtained from the populations were subjected to analysis via R (version 4.2.2). The descriptive statistics were computed, and frequency distribution graphs were plotted. The ANOVA of the traits taken in the study was carried out via the package ‘augmentedRCBD’. The correlations between the traits were also evaluated via SPSS 2.0, along with the significance of the correlations.

### 2.4 β-glucan analysis

Ten-gram seed samples from each of the BC_2_F_2:3_ plants selected on the basis of phenotyping were analysed to determine the grain moisture content, 1,000 kernel weight (TKW), and β-glucan levels. For β-glucan analysis, the sampled grains were finely milled into 0.5 mm particles via a tissuelyser (Retsch MM400). The dry weight basis β-glucan content of the harvested seeds was determined via a β-glucan assay kit (Megazyme, Ireland) specifically designed for mixed linkage (1–3,1–4) β-D-glucan in cereal grains, following the guidelines of the AACC Method 32-23.01. This analysis was performed in triplicate using whole-grain flour. The measurement of the grain moisture content was also conducted in triplicate via a DA 7250 NIR Analyser (Perten, Sweden).

### 2.5 Molecular marker analysis

Leaves from young plants of BC_2_F_2:3_ progenies were used for DNA extraction, followed by the CTAB method of DNA extraction (Murray and Thompson, 1980). The SSR markers covering the entire length of each of the chromosomes in wheat were selected from the wheat genetic maps (Roder et al., 1998; Pestsova et al., 2000; Somers et al., 2004). The selected markers were screened for transferability to *Ae. Kotschyi*. A total of 135 SSR markers were selected on the basis of the screening for transferability. These markers, which exhibit polymorphisms between *Aegilops kotschyi* accession AK-3790 and the recipient wheat cultivars, were used for molecular marker analysis of the BC_2_F_2:3_ lines. In addition to these markers, new primers have also been designed for chromosome 7. The details of the designed primers are given in Online Resource 1. These SSR markers were utilized to validate the presence of *Ae. Kotschyi* chromosomal introgression in situations where plausible candidate chromosome(s) were detected.

### 2.6 Genomic *in situ* hybridization

The genomic DNAs extracted from *Ae. Longissima* (S^l^S^l^) and *Ae. Umbellulata* (UU) were employed to generate genomic probes for use in genomic *in situ* hybridization (GISH) experiments. Actively growing root tips from germinating seeds were subjected to a 24-h treatment with ice water to accumulate metaphases. The root tips were subsequently fixed in a mixture of 3 parts ethanol and 1 part glacial acetic acid. Following fixation, the root tips were stained with 1% acetocarmine and then gently squashed in a solution containing 45% acetic acid. For the preparation of the genomic probes, sheared genomic DNA (ranging from 0.2 kb to 0.6 kb) from *Ae. Longissima* (S^l^S^l^) and *Ae. Umbellulata* (UU) was utilized. For the preparation of the genomic probe of the S^l^-genome, the genomic DNA of *Ae. Longissima* was labelled with fluorescein-11-dUTP (green) (Roche Applied Science, Indianapolis, IN), whereas for the preparation of the genomic probe of the U-genome, the genomic DNA of *Ae. Umbellulata* was labelled with tetramethylrhodamine-5-dUTP (red) (Roche Applied Science, Indianapolis, IN) through nick translation. The labelled probes were purified via the QIA quick Nucleotide Removal Kit (Qiagen, Valencia, CA). Blocking DNA, consisting of unlabelled sheared genomic DNA from Chinese spring wheat (ranging from 100 bp to 1 kb), was employed at a ratio of 1 ng of labelled probe (S^l^- and U-genomes) to 100 ng of blocking DNA. The hybridization conditions and posthybridization washes followed the protocols outlined by Zhang et al. (2001). To enhance visualization, chromosomes were counterstained with 4,6-diamidino-2-phenylindole (DAPI). The slides were analysed via an Epifluorescence Zeiss Axioimager M1 microscope.

## 3 Results

### 3.1 Trait data analysis

Self-pollination of BC_2_F_1_ plants resulted in the generation of 128 distinct BC_2_F_2_ lines, each exhibiting a wide range of phenotypic characteristics. Various yield-related traits, such as per-plant grain yield, thousand-grain weight, spike length, and productive tiller count, were evaluated, along with resistance to yellow rust. The BC_2_F_2_ lines were selfed to obtain the BC_2_F_2:3_ generation lines. Multiple yield-related traits, such as per-plant grain yield, thousand-grain weight, spike length, and productive tiller count, were evaluated, along with resistance to yellow rust (Online Resource 2.1).

**FIGURE 1:**
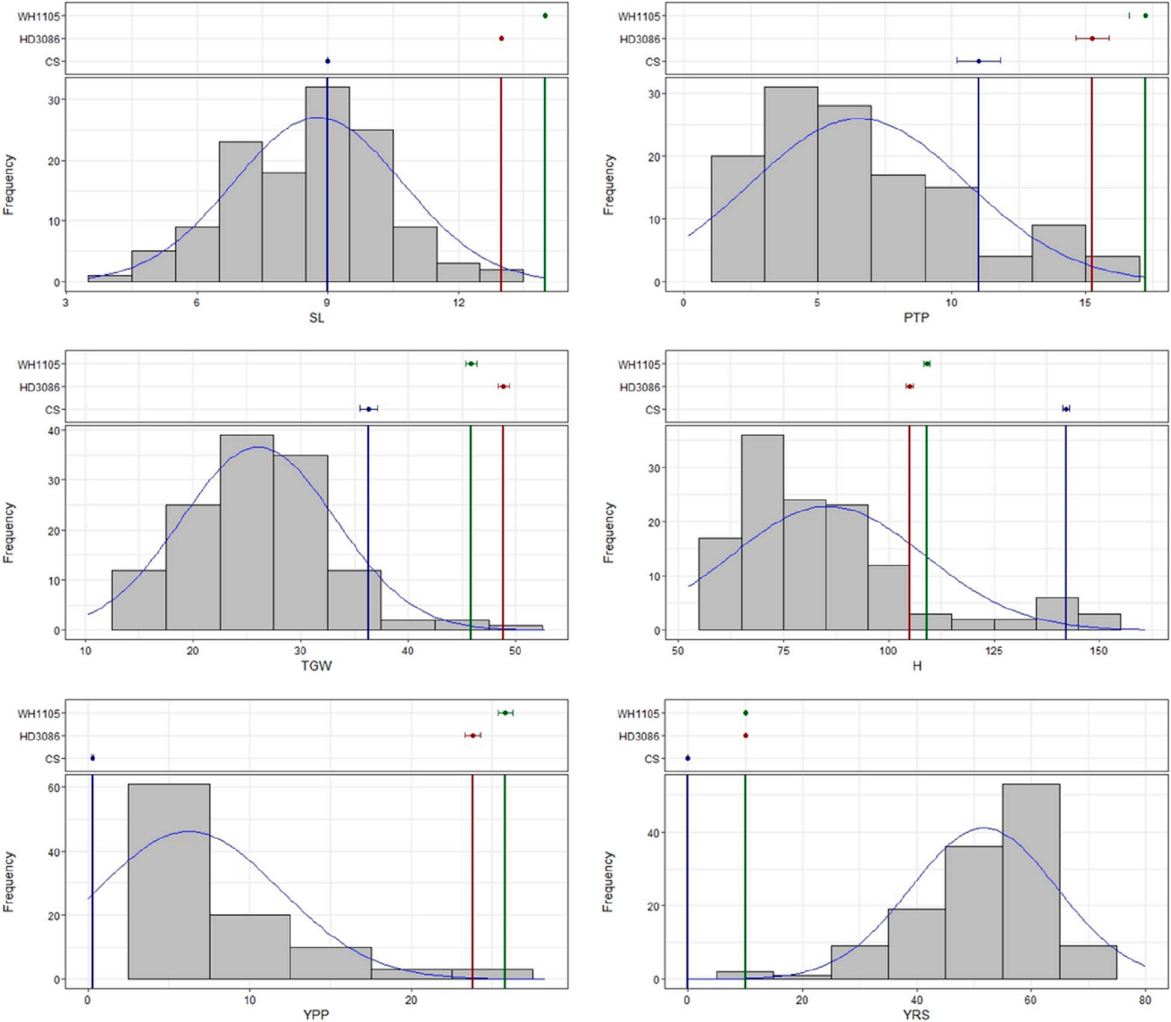
Frequency distributions of the BC_2_F_2:3_ population and parents for the traits spike length (SL), productive tillers/plant (PTP), thousand grain weight (TGW), plant height (H), yield per plant (YPP) and yellow rust severity (YRS).

ANOVA of the 128 BC_2_F_2:3_ genotypes revealed that the variation among the genotypes for each of the traits evaluated in the study was highly significant (Online Resource 2.2). Although the frequency distribution revealed that most of the genotypes presented a relatively low yield per plant, with relatively few productive tillers per plant, a relatively high thousand-grain weight and relatively small spikes, few genotypes presented yield and other yield attributes that were similar to those of the high-yield parents HD3086 and WH1105 (Figure 1). The yield per plant was highly correlated with the number of productive tillers per plant and was significantly negatively correlated with the severity of yellow rust, as expected. Similarly, a significant correlation was observed between thousand grain weight and yield but was negatively correlated with yellow rust infection. Surprisingly, plant height was significantly positively correlated with thousand-grain weight (Table 1).

**TABLE 1:** Simple correlation coefficients (r) between phenotypic traits in the BC_2_F_2:3_ population.

	H	PTP	SL	YPP	YRS
PTP	0.21*				
SL	0.21*	0.05			
YPP	0.18*	0.65**	0.19*		
YRS	−0.01	−0.17	−0.32**	−0.43**	
TGW	0.29*	0.16	−0.07	0.30*	−0.29*

* at 5% level of significance

** at 1% level of significance

These BC_2_F_2:3_ lines were also subjected to meticulous screening, with a focus on various agronomic attributes, including resistance to yellow rust and overall yield potential. During the selection process, desirable parental traits, particularly those associated with high yield, which aim to retain robust yield performance while concurrently inheriting traits related to the β-glucan content in seeds, were also considered. Following a comprehensive disease severity evaluation, the BC_2_F_2:3_ lines were given a disease score of 3 according to the modified Cobb scale of 1–5 provided by Peterson et al. (1948), exhibiting a severity percentage of <60%, displaying a moderate level of tolerance to yellow rust, and a moderate to high per plant yield (≥10.0 g) was prioritized for further analysis. Specifically, this subset of plants (Online Resource 3) was chosen for the estimation of grain β-glucan content, as these criteria ensured a balanced combination of disease resistance and favorable yield potential.

### 3.2 Grain β-glucan

The estimation of β-glucan content in seeds was conducted on a selection of 18 BC_2_F_2:3_ plants chosen on the basis of their distinct phenotypic traits. The data on the β-glucan concentration in the grains were collected in triplicate, and their means were computed to ensure accuracy. Notably, the majority of the plants subjected to β-glucan content analysis presented higher levels of β-glucan content than did the three parental reference strains, namely, Chinese spring (0.77%), WH1105 (0.75%), and HD3086 (0.71%). Among these analysed lines, 9 lines whose β-glucan content exceeded 0.9% are listed in Table 2. Interestingly, one specific line, designated BC_2_F_2:3_ 23-5, presented a notably elevated β-glucan content (1.77%), which surpassed the levels observed in both the other lines and the wheat parental references. However, it is important to note that while this content was significantly high, it did not reach the same level as that found in *Ae. Kotschyi*. Given this remarkable finding, further molecular analysis was initiated for the 9 BC_2_F_2:3_ lines whose β-glucan content exceeded 0.9% to investigate potential introgression from *Ae. Kotschyi*, which might provide insights into the observed high β-glucan content.

**TABLE 2:** Phenotypic parameters of BC_2_F_2:3_ plants with high grain β-glucan contents (≥0.9% dwb).

Line ID	Line pedigree	Height (cm)	Productive tillers/plant	Grain yield/plant (g)	Y rust score (%)	1,000 grain weight (g)	Grain β-glucan content
Recipient Parent	Chinese spring (Ph^ɪ^)	142	11	0.213	NA	36.3	0.77
Donor Parent	*Aegilops kotschyi* 3790	33	112	NA	NA	12.32	2.4
Control 1	*Triticum aestivum* cv. WH1105	109	17	25.17	10	45	0.75
Control 2	*Triticum aestivum* cv. HD3086	105	15	23.15	10	48	0.71
BC_2_ F_2:3_ 21-4	CS (*Ph* ^*ɪ*^)/*Aegilops kotschyi* 3790//WH1105–2P///HD3086-21-4	67	6	13.19	50	31.92	0.90
BC_2_ F_2:3_ 22-5	CS (*Ph* ^*ɪ*^)/*Aegilops kotschyi* 3790//WH1105-2P///HD3086-22-5	72	13	18.77	50	28.66	0.93
BC_2_ F_2:3_ 22-6	CS (*Ph* ^*ɪ*^)/*Aegilops kotschyi* 3790//WH1105-5///HD3086-22-6	54	12	14.3	30	24.75	1.02
BC_2_ F_2:3_ 23-5	CS (*Ph* ^*ɪ*^)/*Aegilops kotschyi* 3790//WH1105-3///HD3086-23-5	140	13	17.02	50	45.40	1.76
BC_2_ F_2:3_ 25-2	CS (*Ph* ^*ɪ*^)/*Aegilops kotschyi* 3790//WH1105-5///HD3086-25-2	91	17	23.95	40	28.42	1.01
BC_2_ F_2:3_ 27-7	CS (*Ph* ^*ɪ*^)/*Aegilops kotschyi* 3790//WH1105-8///HD3086-27-7	62	9	15.21	50	22.35	0.91
BC_2_ F_2:3_ 32-11	CS (*Ph* ^*ɪ*^)/*Aegilops kotschyi* 3790//WH1105-13P///HD3086-32-11	98	7	12.01	50	21.05	0.92
BC_2_ F_2:3_ 32-15	CS (*Ph* ^*ɪ*^)/*Aegilops kotschyi* 3790//WH1105-13P///HD3086-32-15	90	10	15.85	30	27.92	0.92
BC_2_ F_2:3_ 67-7	CS (*Ph* ^*ɪ*^)/*Aegilops kotschyi* 3790//WH1105-16///HD3086-67-7	98	9	14.48	50	28.50	0.90

### 3.3 Molecular analysis

**FIGURE 2:**
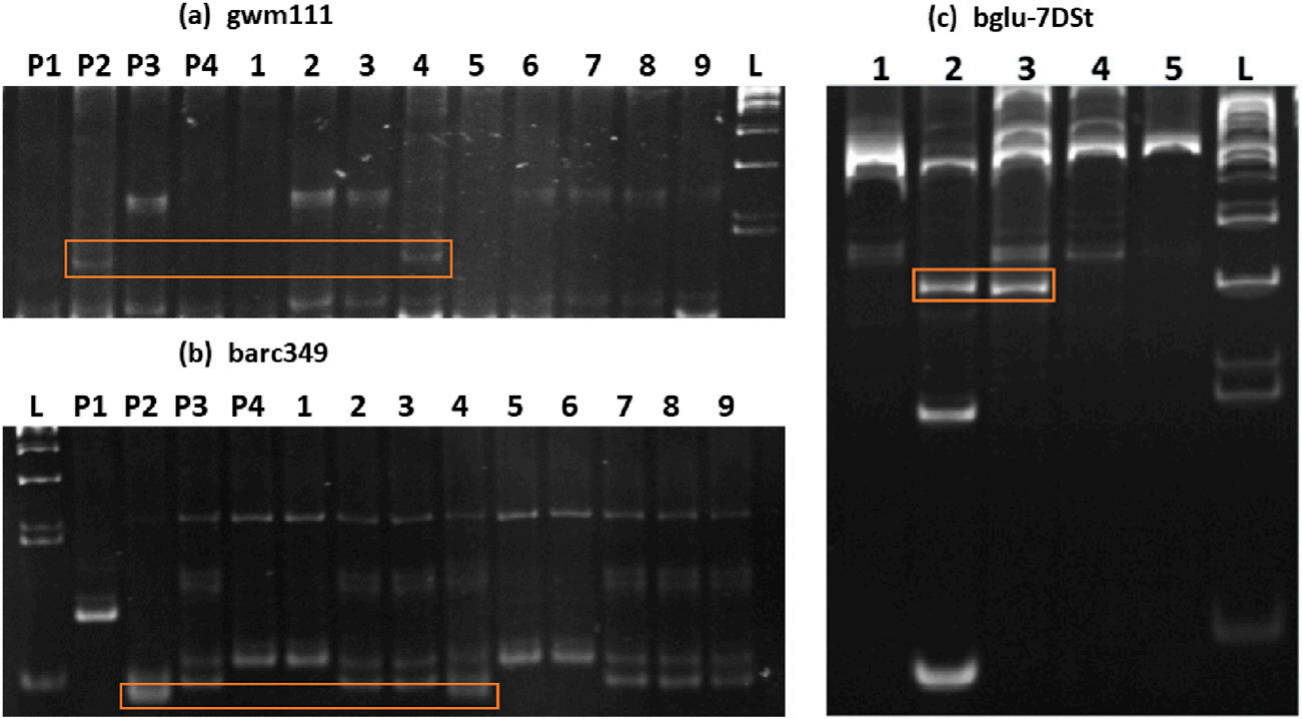
10% urea-PAGE of markers showing introgression of genomic regions from *Ae. Kotschyi* to the progeny BC_2_F_2:3_ line 23-5. The bands that are specific to *Ae. Kotschyi* and BC_2_F_2:3_23-5 are marked. The wells for the SSR markers gwm111 **(a)** and barc349 **(b)** were numbered P1- Chinese spring *Ph* ^*I*^, P2- *Ae. Kotschyi*, P3- WH1105, P4- HD3086, and 1- (21-4), 2- (22-5), 3- (22-6), 4- (23-5), 5- (25-2), 6- (27-7), 7- (32-11), 8- (32-15), 9- (67-7) and L- 50 bp ladders. The wells for the gene specific marker bglu-7DSt **(c)** were numbered as follows: 1- Chinese spring *Ph* ^*I*^, 2- *Ae. Kotschyi*, 3- BC_2_F_2:3_23-5, 4- WH1105, 5- HD3086, and L- 100 bp ladder.

The BC_2_F_2:3_ lines were investigated because of their suspected involvement in carrying vital segments related to high β-glucan content in the *Ae. Kotschyi* genome. To explore this further, a comprehensive molecular marker analysis was performed at the whole-genome level. These molecular markers, which spanned all chromosomes, were amplified via DNA isolated from various sources: Chinese spring (CS), *Ae. Kotschyi*, WH1105, HD3086 and the 9 BC_2_F_2:3_ progenies. The amplified products were then meticulously examined via 10% urea-PAGE to ensure the precise resolution of the amplicons. Verification of *Ae. Kotschyi* genetic introgression into one of the BC_2_F_2:3_ lines, namely, 23-5, was achieved through specific molecular markers, namely, gwm111 and barc349. These markers exhibited variations in both *Ae. Kotschyi* and BC_2_F_2:3_ 23-5 in comparison with those in CS, WH1105, and HD3086 (see Figures 2a, b). Notably, the marker locations were precisely identified on the 2B and 7D chromosomes. These findings confirmed that the introduction of the group 2 and 7 chromosomes originated from *Ae. Kotschyi* into the wheat-*Ae. Kotschyi* derivative line BC_2_F_2:3_ 23-5. To delve deeper into the analysis, selected markers from both arms of these chromosome centromeric and telomeric regions were utilized. This analysis covered the parent lines as well as the derivative BC_2_F_2:3_ 23-5. An important observation was made: a specific primer, bglu-7DSt, originating from the telomeric region of the 7D chromosome’s short arm, displayed variations in both *Ae. Kotschyi* and BC_2_F_2:3_ 23-5 (Figure 2c). This significant insight further confirmed the introgression of the group 7 chromosome from *Ae. Kotschyi* (Online resource 4).

### 3.4 Genomic *in situ* hybridization

**FIGURE 3:**
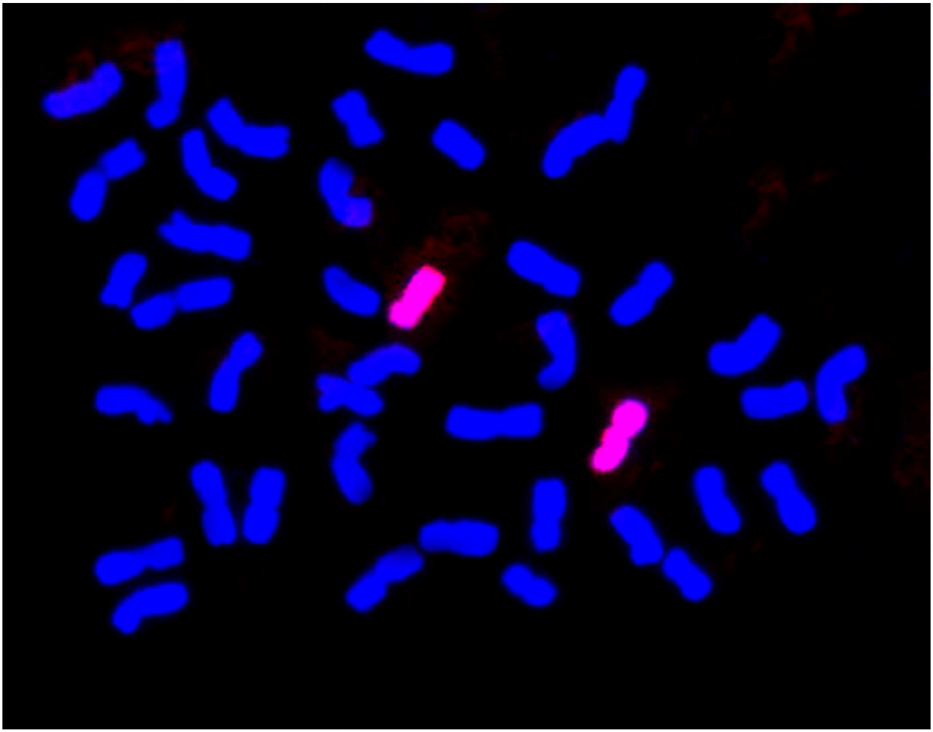
Genomic *in situ* hybridization of BC_2_F_2:3_ 23-5 showing introgression from *Ae. Kotschyii* chromosome 7U.

To increase our understanding of the wheat-*Ae. Kotschyi* derivative line BC_2_F_2:3_ 23-5, in which the integration of group 2 and 7 chromosomes (either 2S, 2U, 7U or 7S) from *Ae. Kotschyi* was previously validated through molecular marker analysis, we employed genomic *in situ* hybridization (GISH). The primary goal of GISH is to provide conclusive evidence regarding the genome of the introgressed chromosome. For the GISH experiments, genomic probes specific to the S and U genomes were employed. Notably, the genomic probe derived from *Ae. Umbellulata* genomic DNA (U-genome) generated a robust hybridization signal on a specific pair of chromosomes of the BC_2_F_2:3_ 23-5 line (Figure 3). This significant hybridization outcome confirmed the presence of the 7U chromosome from *Ae. Kotschyi* in the BC_2_F_2:3_ 23-5 line.

## 4 Discussion

The majority of cultivated bread wheat (*T. aestivum* L.), durum wheat (*T. turgidum* L. ssp. durum), are known to possess relatively low levels of β-glucan in their grain composition. The β-glucan content in bread wheat was less than 1% on a dry weight basis. In contrast, tetraploid wheat genotypes presented a broader range of β-glucan contents, ranging from 0.39% to 0.7% (McCartney et al., 2005). Therefore, in this study, we conducted a thorough investigation into the intricate processes and molecular mechanisms involved in the successful integration of *Ae. Kotschyi* chromosomes. This integration represents a parallel endeavor aimed at cultivating lines inherently equipped to accumulate elevated levels of β-glucan within wheat grains.

Numerous studies have consistently highlighted that the distant relatives of wheat contain notably greater amounts of β-glucan in their grains than cultivated wheat does. Among these wild relatives of wheat, certain members of the *Aegilops species*, specifically *Ae. Biuncialis*, *Ae. Umbellulata*, and *Ae. Neglecta,* presented remarkably elevated β-glucan contents, reaching values as high as 7.1% (Marcotuli et al., 2019). Furthermore, research conducted by (Rakszegi et al., 2017) supported this trend, confirming considerably higher β-glucan contents across all accessions of *Aegilops* sp. In comparison with cultivated wheat genotypes. Specifically, grain β-glucan levels varied within the range of 3%–5% for *Ae. Biuncialis* and *Ae. Geniculate*. Intriguingly, introducing chromosome pairs of 5U^g^, 5 M^g^ and 7 M^g^ from *Ae. Geniculate* and 5U^b^, 5M^b^ and 7M^b^ from *Ae. Biuncialis* into the bread wheat genotype Chinese spring resulted in a noteworthy increase in the β-glucan content (Rakszegi et al., 2017). In this study, we similarly tried to introduce chromosomes from another wild genotype of wheat, *Ae. Kotschyi*, to improve the β-glucan content in wheat while sustaining the increased productivity of the wheat cultivars.

The findings of this study aligned with earlier reports, where they confirmed the introgression of chromosome 7U from *Ae. Kotschyi* in one of the parental derivative lines (63-2–13) of BC_2_F_2:3_ 23-5, which also presented elevated grain Fe and Zn contents (Tiwari et al., 2010). Furthermore, these results are in agreement with those of previous studies (Marcotuli et al., 2017) that identified three significant QTLs for β-glucan content located on chromosomes 2A and 2B. These QTLs were identified through the analysis of recombinant inbred lines (RILs) derived from the Duilio × Avonlea durum wheat cross. Collectively, this body of research emphasizes the likelihood that several genomic regions associated with β-glucan accumulation in wheat grains are predominantly situated within chromosomes 2 and 7. Similarly, a noteworthy QTL on chromosome 7H in barley, which is responsible for 39% of the β-glucan content variation in barley grain (Li et al., 2008), further supports the influence of chromosome seven on the grain β-glucan content. This was evident when the 7H chromosome from barley was introduced into the wheat genome, resulting in a substantial increase in the β-glucan content in wheat grains (Colasuonno et al., 2020). Additionally, Manickavelu et al. (2011) identified β-glucan content QTLs on chromosomes 3A, 1B, 5B, and 6D in wheat via RILs derived from a hybrid between Chinese spring and spelt wheat. Similarly, Marcotuli et al. (2016) employed an array of 230 tetraploid wheat genotypes to predict β-glucan content QTLs. These findings revealed QTLs on chromosomes 1A, 2A, 2B, 5B, and 7A while also identifying a subset of putative genes exerting either direct or indirect influences on the grain β-glucan content. This emphasis on chromosomes 2 and 7 reinforces their pivotal role in β-glucan accumulation, which is consistent with earlier research.

Various yield-related attributes, such as yield per plant, thousand-grain weight, and the number of productive tillers, were considered alongside the parental characteristics of the high-yield parents to ensure that yield levels were maintained while passing through the trait related to the β-glucan content in the seeds. During the BC_1_F_1_ generation, most of the plants were tall. However, within the BC_2_F_2_ and BC_2_F_2:3_ populations, notable variation in plant height was evident. Some plants grew exceptionally tall, even surpassing the height of the Chinese spring parent, whereas a smaller portion of plants presented shorter stature.

Similar diversity was observed for other traits, including resistance to yellow rust, yield per plant, number of productive tillers, and grain β-glucan content. Some plants presented exceptionally high yields and tiller numbers, comparable to or even exceeding those of the high-yield parents HD3086 and WH1105. Conversely, some plants were highly susceptible to yellow rust, in contrast to the parents, whereas others exhibited nearly complete resistance. This wide range of traits among the progeny suggests that these characteristics may be controlled by loci present in the chromosomes introgressed from *Aegilops kotschyi* (Marais et al., 2005). The study of yellow rust resistance revealed a diverse spectrum of infections, ranging from highly susceptible to highly resistant plants within the population, which primarily consisted of landraces. This diversity underscores the quantitative nature of yellow rust resistance, as elucidated by Vikram et al., 2021. In the case of our BC_2_F_2:3_ population, differences among the progenies can be attributed to the segregation of parental genotypes resulting from genetic recombination. Morphological traits, which are inherently quantitative in nature, exhibit variability in their expression due to the combination of different genes and alleles distributed throughout the genome, including the introgressed chromosomes, as observed in our population.

Said et al. (2021) reported that *Ae. Umbellulata* is composed of two significant translocations originating from the short arm of the group 7 chromosome of wheat to chromosome 7UL, followed by a paracentric inversion. Additionally, the second translocation originated from the wheat group 3 short arm and relocated to the 7UL chromosome. The presence of a QTL on chromosome 3 A in wheat has also been reported (Manickavelu et al., 2011). It is feasible that the translocation of wheat group 3 chromosomes could lead to the duplication of genes involved in β-glucan synthesis. This duplication could contribute to increased β-glucan content in wild wheat relatives containing the 7UL chromosome, such as *Ae. Umbellulata*, *Ae. Kotschyi*, and others, including the addition/substitution line generated in this study, namely, 23-5. Importantly, while the U genome of *Ae. Kotschyi* has experienced significant diversification, it still retains similarities to the D genome of wheat (Kilian et al., 2011). This similarity poses challenges in finding SSR markers that exhibit polymorphism, making it difficult to distinguish between these genomes without relying on highly dense markers.

The newly developed *Ae. Kotschyi* introgressed line generated in this study carries a significant amount of unwanted linkage drag. This unintended genetic baggage has led to reduced yield and heightened vulnerability to yellow rust disease, possibly stemming from the disruption of genes responsible for yellow rust resistance. To address these issues, this genotype could be employed in future breeding efforts by initiating a backcrossing program with high-yielding and yellow rust-resistant varieties. This process should involve careful consideration of the transfer of specific chromosomal segments, which can be facilitated through techniques such as irradiation and recombination between homeologous chromosome pairs.

In summary, this study successfully demonstrated the introgression of *Ae. Kotschyi* chromosomes into wheat cultivars, resulting in significantly elevated grain β-glucan contents. Confirmation of this introgressed region was achieved through the use of two SSR markers, gwm111 and barc349, as well as one gene specific marker, bglu-7DSt. Notably, these markers are located on chromosomes two and seven of the wheat genome and are known to harbor QTLs related to the grain β-glucan content. The findings of this research offer valuable insights into the mechanism governing β-glucan synthesis and deposition in wheat grains. This knowledge can serve as a foundation for further analyses and predictions in this field. Moreover, the information presented in this study can be harnessed for future breeding programs aimed at developing wheat varieties with high β-glucan contents. These programs could involve backcrossing the lines established in this study with high-yielding and rust-resistant cultivars, paving the way for the creation of improved wheat varieties with enhanced nutritional profiles.

## Data Availability

The original contributions presented in the study are included in the article/Supplementary Material, further inquiries can be directed to the corresponding author.
